# Spatial Organization of Proteasome Aggregates in the Regulation of Proteasome Homeostasis

**DOI:** 10.3389/fmolb.2019.00150

**Published:** 2020-01-09

**Authors:** Ofri Karmon, Shay Ben Aroya

**Affiliations:** The Mina and Everard Goodman, Faculty of Life Sciences, Bar-Ilan University, Ramat Gan, Israel

**Keywords:** proteasome, PSGs, HSP42, proteaphagy, protein quality control (PQC), insoluble protein deposit (IPOD), CytoQ

## Abstract

Misfolded proteins and insoluble aggregates are continuously produced in the cell and can result in severe stress that threatens cellular fitness and viability if not managed effectively. Accordingly, organisms have evolved several protective protein quality control (PQC) machineries to address these threats. In eukaryotes, the ubiquitin–proteasome system (UPS) plays a vital role in the disposal of intracellular misfolded, damaged, or unneeded proteins. Although ubiquitin-mediated proteasomal degradation of many proteins plays a key role in the PQC system, cells must also dispose of the proteasomes themselves when their subunits are assembled improperly, or when they dysfunction under various conditions, e.g., as a result of genomic mutations, diverse stresses, or treatment with proteasome inhibitors. Here, we review recent studies that identified the regulatory pathways that mediate proteasomes sorting under various stress conditions, and the elimination of its dysfunctional subunits. Following inactivation of the 26S proteasome, UPS-mediated degradation of its own misassembled subunits is the favored disposal pathway. However, the cytosolic cell-compartment-specific aggregase, Hsp42 mediates an alternative pathway, the accumulation of these subunits in cytoprotective compartments, where they become extensively modified with ubiquitin, and are directed by ubiquitin receptors for autophagic clearance (proteaphagy). We also discuss the sorting mechanisms that the cell uses under nitrogen stress, and to distinguish between dysfunctional proteasome aggregates and proteasome storage granules (PSGs), reversible assemblies of membrane-free cytoplasmic condensates that form in yeast upon carbon starvation and help protect proteasomes from autophagic degradation. Regulated proteasome subunit homeostasis is thus controlled through cellular probing of the level of proteasome assembly, and the interplay between UPS-mediated degradation or sorting of misfolded proteins into distinct cellular compartments.

## Introduction

Protein homeostasis encompasses all aspects of a cell's requirements for coordinating protein synthesis, folding, conformational states, localization, stoichiometry of complexes, subcellular distribution, and proteome degradation (Powers et al., 2009; Sontag et al., 2017; Klaips et al., 2018).

Maintaining protein homeostasis is vital to cells as the accumulation of misfolded proteins and the formation of insoluble aggregates can be toxic, and ultimately may even induce cell death. Indeed, the presence of protein aggregates is characteristic of various aggregation diseases such as multisystem proteinopathy (Watts et al., 2004; Brandmeir et al., 2008), and Amyotrophic Lateral Sclerosis (ALS) (Johnson et al., 2010). Cells therefore evolved several protective protein quality control (PQC) machineries that survey proteins both during and after synthesis to detect potentially harmful misfolded proteins, prevent their aggregation, promote refolding, and target those that are terminally misfolded to proteolytic degradation (Chen et al., 2011; Shaid et al., 2013; Willmund et al., 2013; Mogk and Bukau, 2017). Eukaryotic PQC degradation is generally mediated by the ubiquitin-proteasome system (UPS) or by autophagy. While the selective degradation of soluble proteins is usually conducted by the UPS (Finley, 2009), tightly folded proteins, which are resistant to proteasomal degradation, are recognized by the autophagy pathway (Mizushima et al., 2011).

The UPS is a highly conserved 2.5-MD multi-subunit complex that catalyzes the degradation of a large fraction of intracellular soluble proteins (Hershko and Ciechanover, 1998; Finley, 2009; Bhattacharyya et al., 2014). It is comprised of a 20S cylindrically shaped proteolytic core particle (CP), and one or two 19S regulatory particles (RP) that are further divided into lid and base complexes (Coux et al., 1996). The RP together with the CP form the proteasome holoenzyme complex and are localized primarily in the nucleus (Tanaka, 2009; Enenkel, 2014a,b). Ubiquitin–Proteasome System substrates are first tagged with a poly-ubiquitin chain through an enzymatic cascade mediated by enzymes known as E1, E2, and E3 family members. The poly-Ub tag facilitates protein recognition and degradation by the 26S proteasome (Fredrickson and Gardner, 2012; Kästle and Grune, 2012; Amm et al., 2014). In contrast, autophagy is uniquely designed to eliminate larger structures, which are encapsulated, and delivered in bulk from the cytoplasm to either vacuoles (plants and fungi) or lysosomes (mammals) for breakdown and disposal (Reggiori and Klionsky, 2013; Klionsky and Schulman, 2014).

The regulated aggregation of misfolded proteins by chaperones with aggregase activity was recently described as an additional proteostasis strategy (Escusa-Toret et al., 2013; Kumar et al., 2016; Sontag et al., 2017). This pathway was described following the identification of specific deposition sites for misfolded proteins in a variety of cells (Johnston et al., 1998; Kaganovich et al., 2008), suggesting that aggregation is an organized process. Several classes of protein aggregates were identified in the yeast *Saccharomyces cerevisiae*, which are referred to using non-uniform nomenclature. Here, we will use the nomenclature that was recently defined by Bukau and colleagues (reviewed in Mogk and Bukau, 2017). Aggregates that form in the cytosol as a result of proteotoxic stress, under the control of the compartment-specific aggregase, Hsp42 (see below), were originally termed cytosolic aggregates (Specht et al., 2011), stress foci (Spokoini et al., 2012), or Q-bodies (Escusa-Toret et al., 2013); here, we will refer to them as CytoQ. For the large aggregate that localizes next to the vacuole, and was originally described as the deposition site for amyloidogenic proteins, including the yeast prions Rnq1 and Sup35, we will use the original term, the insoluble protein deposit (IPOD) (Kaganovich et al., 2008; Kumar et al., 2016).

The family of small heat shock proteins (sHsps), which exhibit ATP independent chaperone activity, plays an important role in orchestrating the aggregation of misfolded proteins. During unfolding stress, the *S. cerevisiae* Hsps, Hsp42, and Hsp26, associate with substrates in a partially unfolded intermediate state, maintaining them in a ready-to-refold conformation close to the native structure (Haslbeck et al., 2004, 2005). Hsp42 co-aggregates with diverse misfolded substrates under different stress conditions, including heat stress (Specht et al., 2011), proteasome inhibition (Peters et al., 2015, 2016; Marshall et al., 2016), cellular quiescence (Liu et al., 2012), and cellular aging (Saarikangas and Barral, 2015; Lee et al., 2018). Such co-aggregation is employed to actively control the formation of CytoQs and promote the coalescence of multiple small CytoQs into a small number of assemblies of larger size at specific cellular sites (Specht et al., 2011; Escusa-Toret et al., 2013). Substrate sequestration at CytoQs can facilitate their subsequent refolding by ATP-dependent Hsp70-Hsp100 disaggregating chaperones, for subsequent triage between the refolding, and degradation pathways (Mogk and Bukau, 2017).

Since the proteasome is vital for maintaining proteostasis as a part of the PQC, it is involved in nearly all cellular processes. Therefore, elucidating the mechanisms of proteasome turnover and its consequences are of major importance and significance in understanding human diseases caused by protein aggregation (aggregation pathologies).

**Figure 1:**
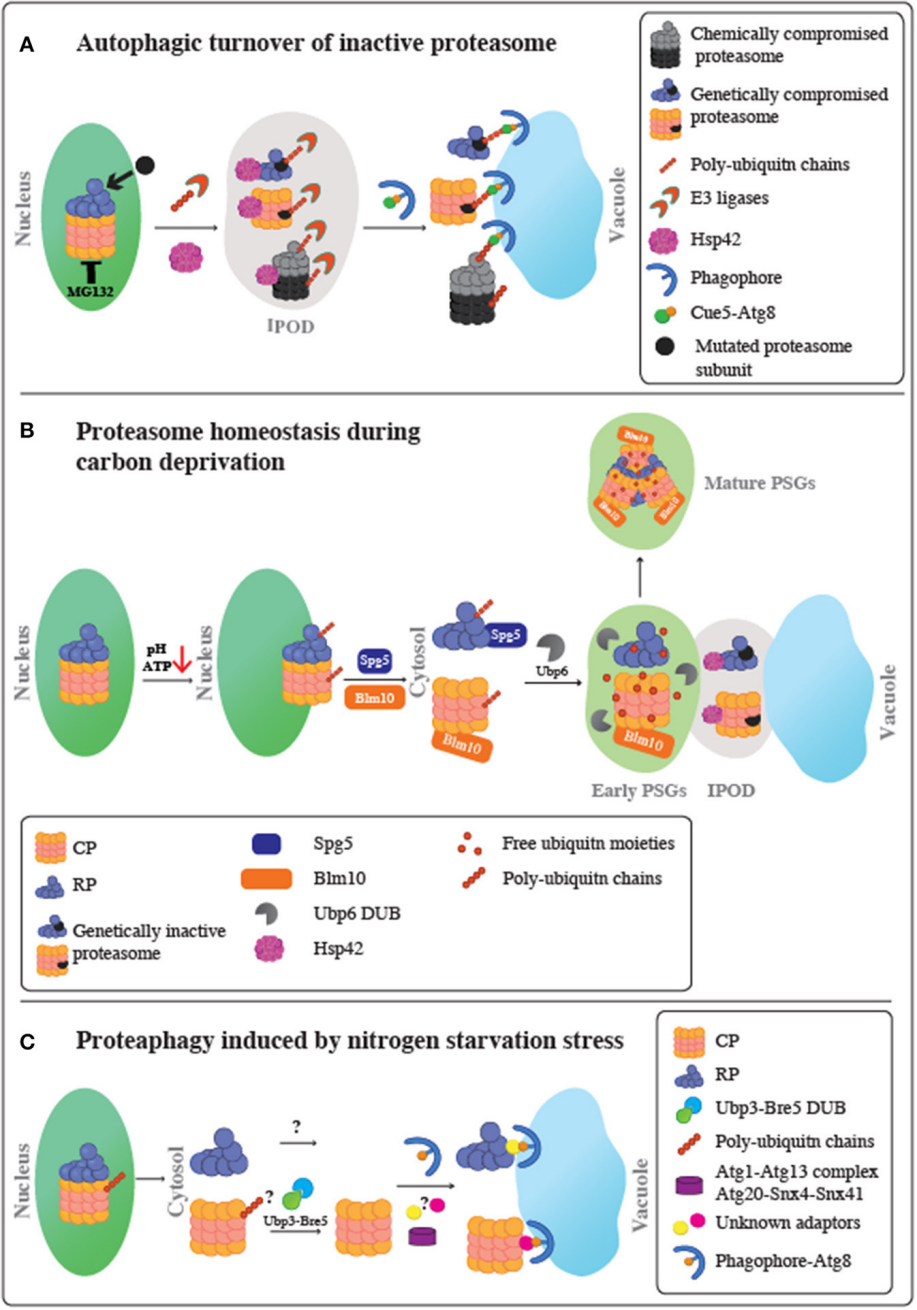
Schematic representation of proteasome fate under various stress conditions. **(A)** Autophagic turnover of inactive proteasome. Following proteasome inactivation, Hsp42 mediates the accumulation of inactive subunits at the IPOD. Proteasomes also become extensively modified with poly-ubiquitin chains in a process mediated by an as yet unidentified E3 Ub ligase. Moreover, it remains unclear whether this ubiquitination step occurs before or after entry to the IPOD. Ubiquitinated proteasomes then associate with the ubiquitin receptor, Cue5, which simultaneously binds to Atg8, leading to their targeting to the autophagic membrane, and proteophagy. Chemical inactivation of proteasomes using the reversible proteasome inhibitor, MG132, stimulates autophagy of both the core particles (CP) and regulatory particles (RP) at similar rates. A genetically compromised RP subunit did not stimulate proteophagy of the CP, and the other way around. Thus, proteaphagy is not restricted to the holo-complex, and RP or CP can be degraded individually. **(B)** Proteasome homeostasis during carbon deprivation. Upon glucose starvation, intracellular ATP levels and pH decrease. This triggers the dissociation of the proteasome holo-complex to CP and RP subcomplexes, migration to the nuclear periphery and a stepwise export from the nucleus to the cytoplasm to form PSGs, membrane-less assemblies of soluble proteins. The first step in the CP and RP cytoplasmic delivery is mediated by Blm10 and Spg5, respectively. This step results in transient association of proteasomes with the IPOD, together with other IPOD proteins, such as Hsp42, to form inclusions termed early PSGs. While mutated inactive proteasomes are retained in these inclusions, the functional CP and RP particles are targeted to form the mature PSGs, which protects functional proteasomes from autophagy. Mature PSG assembly requires the proteasome associated protease, Ubp6, to release free ubiquitin from branched ubiquitin chains on nuclear proteasomes, resulting in a compact granule containing RP, Blm10-CP, and free ubiquitin moieties. This process is reversible; when glucose again becomes available, PSGs disperse, allowing cells to quickly re-enter the cell cycle without waiting for new *de novo* proteasome assembly. **(C)** Proteaphagy induced by nitrogen starvation stress. Upon nitrogen starvation, similarly to carbon depletion, proteasomes are exported from the nucleus to the cytoplasm most likely when the holo-complex is dissociated to CP and RP complexes. Then, each RP and CP is separately targeted to the Atg8-autophagosomes for vacuolar degradation. This process may involve as yet unknown autophagy adaptors and signals. For the CP complex, the role of ubiquitination is still unclear; however, the Ubp3-Bre5 deubiquitinase complex seems to be involved. This autophagy pathway is distinct from the pathway required for inactive proteasomes, since it does not involve Cue5 or Hsp42 proteins. This route depends on Atg1-Atg13 complex, Atg8, Atg9, and Atg15, and the sorting nexins Snx4/Atg24 and Snx41 or Snx42.

Here, we review the important recent advances, and the current stage in our understanding of the principles and mechanisms by which these PQC regulatory pathways regulate the spatial organization or elimination of proteasome subunits under various conditions (see Figure 1 for schematic representation of these pathways).

## Autophagic Turnover of Inactive Proteasome

Proteasomes assemble into very stable complexes (Hendil et al., 2002; Pack et al., 2014); nevertheless, cells need to dispose of them when their subunits are assembled improperly as a result of transcriptional and translational failures, or dysfunction under various conditions, e.g., as a result of genomic mutations, diverse stress conditions such as damage induced by oxidation, or treatment with proteasome inhibitors, such as those now widely used to treat multiple myeloma and other malignancies (Goldberg, 2012). Moreover, non-functional proteasomes can also form during their own assembly, an error-prone process that requires the coordinated activity of numerous assembly chaperones (Enenkel et al., 1999; Bedford et al., 2010; De La Mota-Peynado et al., 2013).

Significant progress in understanding the turnover of inactive proteasome and the formation of different proteasome aggregates was achieved in the model organism *S. cerevisiae* (yeast). Proteasome sequestration and degradation are typically monitored using GFP-tagged proteasome subunits in their wild-type or mutated forms (Enenkel et al., 1999; Weberruss et al., 2013; Enenkel, 2014a,b, 2018; Marshall et al., 2015; Peters et al., 2015, 2016; Marshall and Vierstra, 2018a). Such tagged subunits are also commonly used to monitor vacuolar targeting of proteasomes through autophagy, by observing the accumulation of a ~25 kDa band that is recognized by anti-GFP antibody on immunoblots. The appearance of this band (hereafter termed “free GFP”) results from the rapid cleavage of the linker between GFP and the tagged proteasome subunit. The folding state of the GFP protein renders it resistant to rapid vacuolar degradation, resulting in the accumulation of free GFP that can be also easy detected by fluorescence microscopy (Marshall et al., 2015, 2016; Waite et al., 2016).

Both chemical and genetic approaches have been used in yeast to induce proteasome inactivation. For the chemical approach, the reversible proteasome inhibitor MG132 is added to the growth medium (Marshall et al., 2016; Marshall and Vierstra, 2018a). For the genetic approach, various temperature sensitive (ts) mutants [available from yeast ts mutant collections (Ben-Aroya et al., 2008; Li et al., 2011)] are used to affect selected CP or RP subunits at the restrictive temperatures (Marshall et al., 2015, 2016; Peters et al., 2015; Marshall and Vierstra, 2018a).

When proteasomes are inactivated genetically, using an RP ts mutant that can still assemble functional proteasomes under the semi-restrictive temperature, the functional 26S fraction mediates the degradation of the inactive subunits. However, when functional proteasomes in these cells become scarce following treatment with MG132, or transferring the cells to the restrictive temperature, Hsp42 mediates an alternative pathway, characterized by the accumulation of these subunits in the IPOD (Peters et al., 2015). Notably, these aggregates were recently identified as a prerequisite for the subsequent clearance of dysfunctional proteasomes by autophagy (termed proteophagy) (Marshall et al., 2015, 2016; Waite et al., 2016). As a first line of defense, cells subjected to proteasome inhibition by MG132 treatment, upregulate the expression of the proteasome subunit transcription factor, *RPN4* (Xie and Varshavsky, 2001), and accordingly, no significant proteaphagy is detected during the first hours of proteasome inhibition (Marshall et al., 2016). Impairment of autophagy by deleting enzymes required for the attachment of the lipid phosphatidylethanolamine (PE) of the autophagosomal membrane to Atg8, an essential step in autophagosome formation (Reggiori and Klionsky, 2013), results in the accumulation of GFP-tagged proteasome subunits at the IPOD (Marshall et al., 2016). These results indicate that Hsp42-dependent aggregation occurs upstream to proteophagy. Accordingly, proteaphagy is abolished in Δ*hsp42* cells, and in this case, the GFP-tagged proteasome is no longer observed aggregating in the cytosolic periphery. Instead, it shows the nuclear enrichment similarly to the wt proteasome (Peters et al., 2015, 2016; Marshall et al., 2016). All together, these studies suggest that Hsp42 mediates the coalescence of multiple small CytoQs containing dysfunctional proteasome subunits, into a smaller number of assemblies, until they are sequestered into the IPOD, and that this step is a prerequisite for their delivery to the vacuole and for proteaphagy turnover.

The observation that the proteasome can itself be cleared by autophagy raised several questions, including how the inactivated subunits are recognized and delivered to the autophagic vesicles. Intriguingly, the autophagic clearance of inactive proteasomes requires the induction of their modification with ubiquitin. Several studies suggested that such ubiquitin-modified species or aggregates are recognized by the selective ubiquitin-dependent autophagy machinery (Mizushima et al., 2011; Reggiori and Klionsky, 2013; Lu et al., 2014). The ubiquitin-dependent autophagy pathway plays a key role in the elimination of protein aggregates, assemblies, or organelles, and counteracts the cytotoxicity of proteins linked to neurodegenerative diseases (Klionsky, 2007; Pohl and Jentsch, 2009; Levine et al., 2011). Following substrate ubiquitylation, the ubiquitylated cargo is delivered to autophagosomes through the action of specific adaptors that connect the ubiquitin system with the autophagy pathway. Such adaptors harbor a domain for ubiquitin-conjugate binding, and a distinct binding site for the autophagosomal protein, Atg8 (Kraft et al., 2010). Atg8 becomes conjugated to the lipid phosphatidylethanolamine (PE) of the autophagosomal membrane through a well-characterized conjugation system. This promotes not only docking sites for proteins that induce formation of autophagosomes, but also provides the docking sites for receptors that recruit autophagic cargo prior to their delivery to lysosomal degradation (Levine et al., 2011; Reggiori and Klionsky, 2013). Known adaptors include the human p62/SQSTM1, which carries a domain with ubiquitin-conjugate binding activity (termed UBA), that binds to various ubiquitylated cargo including protein aggregates, pathogens, and peroxisomes, and a distinct binding site for Atg8 (LC3), termed Atg8-interacting motif (AIM), or LC3-interacting region (LIR) (Kirkin et al., 2009; Kraft et al., 2010; Shaid et al., 2013). Another adaptor is the *Arabidopsis* Rpn10 (Marshall et al., 2015), human Tollip, and its functional yeast homolog, Cue5 (Lu et al., 2014; Marshall et al., 2016). Cue5 was first linked to the autophagic clearance of PolyQ aggregates in yeast (Lu et al., 2014). Later, it was demonstrated that Cue5 forms a complex with both the ubiquitinated 26S proteasome through its CUE domain, a ubiquitin binding domain of the CUET proteins, and with Atg8 through its Atg8 -interacting motif (AIM) (Marshall et al., 2016).

Together, these studies suggested that directing the ubiquitinated dysfunctional proteasomes to the IPOD, next to the vacuole, allows Cue5 to deliver the sequestered substrates to the adjacent Atg8, thereby facilitating encapsulation. This provides an important surveillance mechanism for the recycling of inactive proteasomes by proteophagy, and the maintenance of 26S proteasome homeostasis. Interestingly, a genetically compromised RP subunit did not stimulate proteophagy of the CP. In a similar fashion, RP proteophagy was not stimulated under CP damage (Marshall et al., 2016). However, chemical inactivation of the CP peptidase activity with MG132 stimulates autophagy of both the CP and RP at similar rates. Thus, it appears that while proteophagy can eliminate the entire 26S complex, specific damage to the CP or RP can result in their selective removal. The previous observation that the CP and RP are tightly associated upon inhibition of the CP (Kleijnen et al., 2007), provides a possible explanation for the removal of both subcomplexes following MG132 exposure, even though only the CP active sites are compromised.

Conflicting data have been reported regarding the role of ubiquitination in targeting substrates to various deposition sites. It was originally shown that formation of CytoQ containing misfolded model substrates such as VHL and Ubc9ts (Kaganovich et al., 2008), or DegAB-GFP (Alfassy et al., 2013) requires ubiquitination for their sorting. However, in another study, although the misfolded substrate tGnd1-GFP was ubiquitinated, deletion of its E3 ubiquitin ligases had no effect on its *HSP42* dependent sequestration (Miller et al., 2015a). Hence, these studies indicate strong substrate-specific variations in the role of ubiquitination as a required sorting signal. Accordingly, while it is well-established that ubiquitination of the damaged proteasome is conserved both in *Arabidopsis*, and in yeast (Marshall et al., 2015, 2016; Waite et al., 2016), it is not clear whether the non-native conformational state of the dysfunctional proteasome is sufficient to target these structures to the IPOD, via Hsp42, which in turn facilitates their subsequent ubiquitylation through the associated E3s. Alternatively, it is possible that ubiquitin addition is required for Hsp42-mediated aggregation into the IPOD.

Another key question in the proteophagy of inactivated proteasome components is the identity of the E3 ubiquitin ligases that act on these substrates. Co-immunoprecipitation experiments revealed an association between Cue5 and the E3 ubiquitin ligase, Rsp5, the main ubiquitin ligase that targets cytosolic misfolded proteins following heat stress (Fang et al., 2014). Moreover, cell fractionation assays revealed that Atg8 and Cue5 co-precipitate with the poly-Q protein, Htt-96Q, and demonstrated that Rsp5 is required for the clearance of these aggregates by the selective ubiquitin-dependent autophagy pathway (Lu et al., 2014). In an analogous manner, it is possible that Rsp5 may also participate in the ubiquitination of inactive proteasomes. A potential role suggested for Rsp5 in mediating the degradation of misfolded proteins was in priming their ubiquitination, with extension of ubiquitin chains on the conjugated substrates carried out by another E3 elongating enzyme (also termed E4) (Koegl et al., 1999; Fang et al., 2014). Therefore, while Rsp5 is a potential candidate for this ubiquitination step, other E2 or E3 enzymes that mediate proteophagy, are yet to be identified.

Whereas, the regulation of proteasome degradation of damaged proteins has been studied for decades, little was known about the regulation of its own impaired units. In this case, proteaphagy represents a cellular strategy to degrade proteasome components when the UPS system malfunctions. This process does not involve random bulk autophagy, but rather, is a highly regulated process involving the protein quality control machinery via its chaperones and ubiquitin-autophagy adaptors. How the protein quality control mechanism identifies impaired proteasome subunits and distinguishes them from other types proteasome aggregates remains unclear and should be further explored.

## Proteaphagy Induced by Nitrogen Starvation Stress

Proteaphagy is initiated in response to proteasome inactivation or nitrogen deprivation (Marshall and Vierstra, 2015, 2018a,b; Marshall et al., 2015, 2016). However, these pathways are distinct, as they require expression of different autophagy genes (Marshall et al., 2016; Marshall and Vierstra, 2018a). More than 80% of the yeast and Arabidopsis proteasomes are subjected to proteaphagy within the first 8 h of transfer to nitrogen-deprivation conditions (Marshall et al., 2015; Marshall and Vierstra, 2018a). This degradation depends on *ATG1*, a nutrient responsive kinase, *ATG7*, a core autophagy component, and *ATG13*, a regulatory subunit of the Atg1 kinase (Reggiori and Klionsky, 2013; Dikic, 2017; Galluzzi et al., 2017), but not on *CUE5* and *HSP42* (Marshall et al., 2015; Marshall and Vierstra, 2018a). An additional factor that distinguishes nitrogen starvation is the role of the de-ubiquitinase, Ubp3 and its co-factor Bre5. Ubp3 and Bre5 are involved in nitrogen depletion-induced proteaphagy, under which they promote autophagy of the CP, but not the RP (Waite et al., 2016; Marshall and Vierstra, 2018a). Nevertheless, the machinery that induces proteaphagy in response to nitrogen depletion needs to be further investigated in comparison to proteasome inactivation.

## Proteasome Homeostasis During Carbon Deprivation

When glucose is depleted, and ATP levels decrease, the subsequent acidification of the cytoplasm signals cells to enter quiescence, the stationary (G_0_) phase in yeast (Laporte et al., 2008; Munder et al., 2016). This state has been shown to promote widespread-programmed reorganization of nuclear and cytoplasmic proteins into reversible assemblies (Narayanaswamy et al., 2009; Breker et al., 2013). One of these assemblies forms as a result of the massive cytoplasmic re-localization of proteasome subunits into proteasome-storage granules (PSGs) (Bajorek et al., 2003; Laporte et al., 2008; Peters et al., 2013). PSGs are thought to be membrane-less droplets of soluble proteins (Enenkel, 2018). During the first step of PSG formation, proteasomes migrate to the nuclear periphery (Laporte et al., 2008; Daignan-Fornier and Sagot, 2011; Enenkel, 2014a,b). Then, before being deposited in their final cytosolic assemblies, they transiently co-localize with Hsp42 in the IPOD (Peters et al., 2016). PSGs act as cellular reservoirs, which help protect the proteasome pool by its sequestration (Laporte et al., 2008, 2011). When conditions improve, and glucose becomes available again, PSGs disperse, allowing cells to quickly reenter the cell cycle without waiting for the *de novo* synthesis and assembly of new proteasomes (Laporte et al., 2008, 2011; Daignan-Fornier and Sagot, 2011).

While both the CP and RP co-localize in PSGs, studies have shown that they are not assembled with each other during PSG storage (Bajorek et al., 2003; Chowdhury and Enenkel, 2015; Peters et al., 2016). Upon carbon starvation, the CP and RP dissociate and are separately delivered into PSGs, by Blm10, and Spg5, respectively (Decker and Parker, 2012; Hanna et al., 2012; Weberruss et al., 2013; Marshall and Vierstra, 2018a), where instead of an association of the CP with the RP, a large fraction of the CP is seen interacting with Blm10 (Weberruss et al., 2013; Gu et al., 2017). Furthermore, *in vitro* measurement of the proteolytic activity of CP and RP-CP after carbon starvation showed that while CP activity was high, RP-CP activity dropped, reinforcing the contention that CP and RP do not associate under conditions that favor PSG formation (Marshall and Vierstra, 2018a). Still, PSG formation requires the presence of both intact CP and RP, since in cells carrying a mutation in RP subunits, the CP was no longer embedded in the form of PSGs, and was dispersed in the cytoplasm (Saunier et al., 2013; Peters et al., 2016). These results suggest that while PSGs contain intact RP and CP, the holo-complex is not assembled, and is most probably dysfunctional under carbon starvation (Weberruss et al., 2013). This is consistent with the original role suggested for PSGs (Laporte et al., 2008). The ability to rapidly restore proteasome capacity avoids the need to reassemble the proteasome pool *de novo*, which would be essential for proper regulation of cell division and other growth-promoting processes (Laporte et al., 2008). Indeed, PSG formation promotes resumption of cell growth upon exit from starvation, even when the cell is subjected to additional folding stress imposed by the amino acid analogs, canavanine and p-fluorophenylalanine (Marshall and Vierstra, 2018a). Survival under these conditions would be aided by the capacity of proteasomes to clear abnormal proteins incorporating these analogs (Marshall and Vierstra, 2018a).

To test the requirement of free ubiquitin in PSG formation, deletion mutants were made for Ubp6, the proteasome-associated ubiquitin-specific protease that releases free ubiquitin from branched polyubiquitin chains (Crosas et al., 2006; Sakata et al., 2011) and Ubi4, which encodes a penta-repeat of ubiquitin molecules (Finley et al., 1987). Both Δ*ubp6* and Δ*ubi4* cells are characterized by low levels of free ubiquitin (Hanna et al., 2006, 2007). In both mutants, PSG formation was perturbed and the resumption of growth upon exit from quiescence was delayed. These results revealed the importance of ubiquitin in the formation of PSGs (Gu et al., 2017; Enenkel, 2018). It was demonstrated that proteasomes in PSGs are enriched with not covalently attached free ubiquitin, as opposed to proteasomes in proliferating cells, which are enriched with poly-ubiquitin chains (Gu et al., 2017). Furthermore, when tracking the GFP-tagged version of Ubi4 and Ubp6, it was shown that under carbon starvation, they both formed PSG-like foci, which co-localized to PSGs, and cleared upon carbon restoration (Gu et al., 2017). PSG formation can be prematurely induced in proliferating cells by the over production of Ubi4 (Gu et al., 2017). Furthermore, the overproduction of a lysine-free Ubi4 which is catalytically inactive and cannot form any ubiquitin chains, results in premature induction of PSGs. All together, these results support the notion that free ubiquitin levels regulate PSG formation, and that prior to sequestration in PSGs, proteasomes in proliferating cells must trim their ubiquitin branches. The presence of mono-ubiquitin triggers nuclear export and sequestration into PSGs (Gu et al., 2017). The requirement for de-ubiquitylating enzymes to increase free ubiquitin levels, and trigger PSG formation needs to be further studied.

Attempts to identify additional proteins that aggregate similarly to PSGs, and might form a PSG scaffold, were described in two independent studies. Both used an arrayed GFP clone collection of *S. cerevisiae* tagged open reading frames (Huh et al., 2003) to systematically follow the reorganization of yeast proteins under carbon starvation (Gu et al., 2017; Lee et al., 2018). Other than Ubi4, Ubp6, Blm10 and the proteasome subunits themselves, none of the detected protein aggregates were reversable upon carbon addition, or co-localized with PSGs (Gu et al., 2017; Lee et al., 2018). These results suggest that PSGs are unique and represent reservoirs of ubiquitin and proteasome subunits.

## PSGs and Proteaphagy are Mutually Exclusive Proteasome Fates

It has been demonstrated that PSG assembly and proteaphagy are mutually exclusive proteasome fates, and thus, when PSG formation is blocked, proteaphagy occurs (Marshall and Vierstra, 2018a). Carbon starvation, which induces PSGs, selectively suppresses proteaphagy, despite the up regulation of bulk autophagy and other forms of selective autophagy, which are induced under such conditions. In addition, when nitrogen and carbon starvation were combined, lack of carbon had a dominant effect on proteasome fate by rapidly suppressing proteaphagy and promoting PSG formation (Marshall and Vierstra, 2018a). However, it remains unknown why carbon starvation, but not nitrogen starvation, induces PSGs and other protein re-arrangements.

Blm10, which is essential for directing the CP to PSGs, appears to protect the CP from direction to proteaphagy (Marshall and Vierstra, 2018a). The absence of *BLM10* under carbon starvation pushes cells to proteaphagy, which can be blocked on Δ*atg7* and Δ*atg13* backgrounds, but not in Δ*cue5* cells. This indicates that Blm10 blocks the autophagy pathway of the CP through nutrient deprivation proteaphagy rather than proteaphagy pathways induced by inactive proteasomes (Marshall and Vierstra, 2018a).

Table 1 summarizes the conditions and factors found thus far to impact PSG formation, while inversely affecting proteaphagy. Nevertheless, the common master regulator that links these factors in the process of PSG formation remains unknown.

**Table 1:** Conditions that impact PSGs formation inversely affect proteaphagy.

**Promotes PSGs formation**	**Promotes autophagy**	**Effect on RP or CP**	**References**
Acetylase NatB	Δnatb (catalytic subunit)	RP +CP	Van Deventer et al., 2015
	Δmdm20 (regulatory subunit)		
Rpn11—intrinsic deubiquitylase of the RP	Rpn11-m1 Rpn11-m5	RP	Saunier et al., 2013; Marshall and Vierstra, 2018a
Low pH—resembles quiescence using CCCP	High pH	RP +CP	Peters et al., 2013
Energy depletion—reduced ATP using 2-DG		RP +CP	Gu et al., 2017; Marshall and Vierstra, 2018a
Blm10	Δblm10	CP	Weberruss et al., 2013
Spg5	Δspg5	RP	Hanna et al., 2012
Over expression of UBI4		RP +CP	Gu et al., 2017
(Ubp3 is not required for PSGs formation)	Ubp3	CP	Marshall and Vierstra, 2018a

## The Role of Small Heat Shock Proteins in Facilitating Granule Formation

Maintaining proteostasis is crucial for cell function and viability. It is critical for the cell to be able to control the toxic potential of misfolded proteins and aggregates by either sequestration, degradation or both. However, cells must also retain the capacity to regulate the transition between proliferation and quiescence, specifically by protecting “reservoirs” of properly folded protein granules in times of stress. If not balanced, these conflicting pathways can be potentially deleterious for the entire organism.

Hsp42 has been shown to organize the sequestration of diverse substrates under different stress regimes, including heat stress, proteasome inhibition, cellular quiescence, and cellular aging (Liu et al., 2012; Escusa-Toret et al., 2013; Miller et al., 2015a; Peters et al., 2015, 2016; Saarikangas and Barral, 2015; Marshall et al., 2016; Ungelenk et al., 2016). The role of Hsp42 in these processes is specific and cannot be replaced by other chaperones. Hsp42 maintains its functional specificity as a cellular aggregase via its long N-terminal extension (NTE) (Alberti et al., 2009; Jaya et al., 2009; Specht et al., 2011; Fu et al., 2013; Grousl et al., 2018), while NTE deletion abrogates CytoQ formation (Mogk and Bukau, 2017; Grousl et al., 2018). The aggregase function of Hsp42 was suggested to include two distinct activities. First, Hsp42 directly promotes protein aggregation; in addition, Hsp42 mediates the coalescence of multiple small CytoQs into a smaller number of CytoQs of enlarged size (Escusa-Toret et al., 2013; Saarikangas and Barral, 2015; Ungelenk et al., 2016; Mogk and Bukau, 2017; Grousl et al., 2018).

A major, and as yet unresolved question is how Hsp42 recognizes its substrates for aggregation. Is it exclusively based on the exposure of hydrophobic patches on the substrates? Does the solubility state of the substrate affect its binding? Finally, is Hsp42 alerted by the presence of a signal, such as ubiquitination?

Ubiquitination was originally described as a sorting signal for Hsp42-dependant CytoQ formation (Shiber et al., 2013). Moreover, the quality control machinery partitions misfolded proteins to compartments on the basis of their solubility and ubiquitination state (Kaganovich et al., 2008; Spokoini et al., 2012). This suggests that misfolded proteins targeted to degradation or sequestration are ubiquitylated as part of a step prior to aggregation. In support of this pathway, is the demonstration (Shiber et al., 2013) that the Hsp40 co-chaperone, Sis1, is required for the ubiquitylation of proteins carrying the DegAB degron, and in Δ*ssa1/2* cells, they are sequestered into detergent-insoluble, Hsp42-positive inclusion bodies.

Hsp42 is crucial for the assembly of proteins that have a role in epigenetics, metabolic enzymes and molecular chaperones, during the stationary phase. These granules were termed Hsp42-Sationary phase granules (SPGs) (Liu et al., 2012; Lee et al., 2016, 2018), and their presence was shown to promote cell survival during stress (Liu et al., 2012; Lee et al., 2016, 2018). It was also demonstrated that under carbon starvation, Hsp42-SPGs co-immunoprecipitates with Ssa1-TAP and not Hsp42-TAP (Lee et al., 2018). This may indicate that under carbon starvation stress, Hsp42 is predominantly insoluble, while Ssa1 (Shiber et al., 2013) and the other SPG components (Lee et al., 2018), similar to PSGs (Enenkel, 2018), are soluble. Taken together, these results imply that Hsp42 may preferentially sequester insoluble proteins.

In this regard, Hos2, a known component of Hsp42-SPGs (Liu et al., 2012; Lee et al., 2018), behaves under carbon starvation stress in a manner similar to that of luciferase, containing mutations that induce protein misfolding (Lee et al., 2018). The ability of such mutations to induce protein misfolding, raises the possibility that only proteins prone to misfolding are gathered to Hsp42-dependent SPGs (Lee et al., 2018). Furthermore, when SPG components were compared to proteins previously shown to have a tendency to misfold and form aggregates in log phase cells after heat shock (Ruan et al., 2017), 50% of the SPG proteins overlapped (Lee et al., 2018). It is likely that these proteins are protected by molecular chaperones in Hsp42-SPGs to prevent further misfolding and damage during chronological aging or stress (Lee et al., 2018). When yeast cells enter stationary phase, proteasome activity is also gradually decreased (Bajorek et al., 2003); therefore, it is possible that stationary phase cells use Hsp42-SPGs to collect partially misfolded proteins to prevent further damage or perturbation. If this is the case, the presence of a ubiquitination signal on these misfolded proteins can trigger Hsp42 to sequester semi-functional proteins, as well.

Nevertheless, there are two major arguments against the function of ubiquitin as a recognition signal. First, the re-constitution of Hsp42 aggregase activity *in vitro* shows that Hsp42 is necessary and sufficient to promote protein aggregation (Ungelenk et al., 2016; Mogk and Bukau, 2017). Hsp42 acts by increasing the concentration of hydrophobic patches in the substrate (Ungelenk et al., 2016; Mogk and Bukau, 2017), implying that no additional cellular factors are required for CytoQ formation, and indeed no such factors have been reported. Second, it has been shown that the formation of SPGs (Liu et al., 2012; Lee et al., 2016, 2018), and stress granules (Buchan and Parker, 2009; Malinovska et al., 2012, 2013; Protter and Parker, 2016) during the stationary phase is also Hsp42-dependent. Both types of granules harbor intact proteins without any requirement for ubiquitination. Moreover, the intranuclear quality control compartment (INQ), which is a depository for misfolded aggregating proteins, does not require ubiquitination either (Miller et al., 2015a,b). Accordingly, it was suggested that Hsp42 may function as a scaffolding molecule that promotes its own interactions with proteins, and that its NTE, specifically its Prion like Domain, constitutes its major substrate-binding site (Mogk and Bukau, 2017). Further support for this notion comes from the fact that Hsp42 is very abundant at 30°C (28,000 molecules/cell) and even more so during heat-stress (46,000 molecules/cell) (Miller et al., 2015a). This high availability of Hsp42 allows the proteins subjected to aggregation to be constantly exposed to Hsp42 and to interact with it more frequently.

Dysfunctional proteasomes are sequestered to the IPOD in an Hsp42-dependent manner (Peters et al., 2015; Marshall et al., 2016). In contrast, under carbon starvation, the intact CP and RP components embedded in PSGs, only transiently co-localize with Hsp42 at the IPOD. As a result, these components are not retained in the IPOD and are subsequently directed to a distinct inclusion site (Peters et al., 2016). These observations are consistent with the findings demonstrating that proteins prone to misfolding upon heat shock or in stationary phase are sequestered to an Hsp42-associated granule (Lee et al., 2018). The transient association with Hsp42 demonstrates that proteasome sequestration to deposition sites is highly regulated and requires a quality control step (Peters et al., 2016). This regulation ensures that upon carbon depletion, proteasome sub-complexes embedded in PSGs remain intact to support their rapid reassembly and re-entry into the cell cycle, even in the absence of *de novo* protein synthesis (Laporte et al., 2008, 2011). All these observations support the existence of another, as yet unidentified, cellular signal, which promotes the transient association between PSGs and Hsp42.

Both CytoQ and SPG share chaperones, such as Hsp104, Hsp26, Ssa1-4, Ydj1, and Get-complex components (Escusa-Toret et al., 2013; Lee et al., 2018), suggesting that chaperones are the core proteins that interact with additional substrate proteins to form granule structures under distinct stresses. This supports a previous hypothesis that the IPOD serves not only as the well-established sequestration site for terminally misfolded proteins, but also as a functional sorting compartment (Peters et al., 2016). Furthermore, it was shown that Hsp42-SPG formation not only allows cells to regulate protein activities during the stationary phase and its exit to the cell cycle (Liu et al., 2012; Lee et al., 2016, 2018), it also provides a PQC center for cells to respond to sudden stress.

PSGs have been shown to transiently co-localize not only with Hsp42, but also with the IPOD marker, the yeast prion, Rnq1 (Peters et al., 2016). Moreover Hsp42-SPG and Rnq1 were shown to partially overlap (Lee et al., 2018). It is possible that the cellular position of the different types of Hsp42-associated granules and aggregates, SPGs, Hsp42-cytoQ, dysfunctional proteasome in the IPOD, and PSGs in their co-localizing state with Hsp42, are located adjacent to each other and may partially overlap in their position within the cell. Moreover, the fact that they share chaperones including Ssa1-4, Ydj1, Get complex protein, Hsp104 Hsp26, Hsp42 as their components can suggest that the regulation of Hsp42-cytoQ and the IPOD is tightly associated, as described for SPGs (Lee et al., 2018), PSGs (Peters et al., 2016), and dysfunctional proteasomes in the IPOD (Peters et al., 2015). Hsp42 might represent a general quality control hub that broadly impacts proteins that change their localization in response to various types of stress.

## Closing Remarks

The role of aggregation in maintaining the homeostasis of proteasomes and other proteins challenge the traditional view of misfolded protein clearance mechanisms, which were proposed to be hierarchical, i.e., misfolded proteins were believed to be initially stabilized by chaperones for either refolding or degradation, with sequestration into aggregates as an undesired stochastic process resulting from collapse of proteostasis. However, it is now believed that the organized co-aggregation of misfolded protein with sHsps, and their sequestration in large cellular assemblies represents a novel parallel pathway to proteostasis. Many proteins have been shown to serve as substrates for different types of aggregation, in diverse deposition sites, either as intact proteins and complexes, or misfolded proteins. This suggests that the cell must be able to recognize and direct different types of proteins to their respective deposition sites. Moreover, cells may have a sorting center to cope with the different types of aggregation induced under the diverse stress conditions they face. Malfunction of this machinery can be deleterious for the cell. Since most of the different types of aggregates are found in mammalian cells, as well, elucidating the factors that identify and target the different types of protein aggregation will shed light on their potential role.

## Author Contributions

All authors listed have made a substantial, direct and intellectual contribution to the work, and approved it for publication.

### Conflict of Interest

The authors declare that the research was conducted in the absence of any commercial or financial relationships that could be construed as a potential conflict of interest.
